# Experimentally validated finite element model for mechanical and fracture characteristics of SiCN thin films under different loads

**DOI:** 10.1038/s41598-025-15659-5

**Published:** 2025-08-28

**Authors:** Dhruva Kumar, Rajesh Kumar Meena, Hirshikesh, Ranjan Kumar Ghadai

**Affiliations:** 1 Department of Mechanical Engineering, Sikkim Manipal Institute of Technology, Sikkim Manipal University, Majitar, Sikkim, 737136 India; 2 Department of Industrial Engineering, University of Trento, Via Sommarive 9, 38123 Trento, Italy; 3 Department of Mechanical Engineering, Indian Institute of Technology Jodhpur, Jheepasani, Rajasthan 342037 India; 4 Department of Mechanical and Industrial Engineering, Manipal Institute of Technology, Manipal Academy of Higher Education, Manipal, Karnataka 576104 India

**Keywords:** SiCN thin film, Nano-indentation, FE-model, Coating cracking, Interface delamination, Engineering, Materials science

## Abstract

In this work, SiCN thin films were deposited on p-Si (100) substrate using a thermal Chemical Vapor Deposition (CVD) process. The mechanical behavior of the thin film was characterized using the nanoindentation technique, where the load was varied from 1 to 4 mN, to understand the influence of load variation on the load-displacement response. Additionally, an experimentally validated FE model, incorporating an elast-plastic material response of the thin film, was developed to understand localized stress distribution and fracture behavior. The fracture behavior is examined through two modes: (a) cracking and interfacial delamination during the nano-indentation test and (b) the peel test. The FE model revealed that in the case of the weak cohesive interface between SiCN and Si, the interfacial failure initiates at a critical displacement of $\sim$ 110 nm. During the peel test, it was observed that the critical fracture energy of the interface plays a significant role in the interface debonding. These finding highlights the strong dependence of the mechanical integrity of the SiCN thin film on the applied load.

## Introduction

Recently, there has been significant interest in silicon carbo-nitrides (SiCN) thin films for their applications in various fields such as micro-electromechanical systems (MEMS), wear-resistant coating, microelectronics, and optoelectronics. The interest stems from their remarkable mechanical, tribological, and optical properties as well as their chemical inertness ^1^. These coatings offer various advantages in coating technologies, such as good wetting behavior, low coefficient of friction, high-temperature stability, and high oxidation resistance^2–6^. SiCN thin films also hold promise in the fields of microelectronics and optoelectronics due to their unique “tunable” band gap properties, adjustable transparency in the visible and infrared (IR) ranges, and excellent thermal stability^7–9^. The SiCN thin films are synthesized using physical or chemical deposition methods. The commonly used methods of synthesis are low-pressure chemical vapor deposition (LPCVD) ^10,11^, plasma-enhanced chemical vapor deposition (PECVD) ^12,13^, atmospheric pressure chemical vapor deposition (APCVD) ^14^, direct current sputtering (DC sputtering) ^15^, and radio frequency sputtering (RF sputtering) ^16,17^.

However, when thin films are in use, they frequently experience extremely high stresses that can cause distortion, deformation, fracture, degradation, or decohesion of the film due to accelerated diffusion or corrosion^18,19^. Enhancing the reliability and performance of thin films necessitates a deeper comprehension of their mechanical and fracture characteristics ^20,21^. The nanoindentation test is frequently used to examine the mechanical characteristics of coatings at the micro and nanoscales. Nanoindentation has several benefits; it is easy to conduct, restricts the indentation depth to mitigate the substrate effects, and can measure various mechanical parameters like hardness, elastic modulus, fracture toughness, creep, and yield stress at the nanoscale^22,23^. Previous studies on measuring the mechanical properties of bulk metallic glass using nanoindentation have examined the influence of test conditions, including peak load and loading rate^24,25^. These investigations have revealed that the hardness of bulk metallic glass becomes load-independent, while the elastic modulus increases with the applied load^26^. However, the impact of these test conditions on the evaluation of mechanical properties for SiCN thin film has yet to be explored.

In addition, the nanoindentation test cannot provide detailed information on local deformation, stress distribution, and localized stress concentration ^27,28^. To address these limitations, finite element simulation of nano-indentation test has been utilized, which helps to overcome the aforementioned experimental issues ^29–33^. By employing the finite element method (FEM), the experimental load indentation depth response can be easily reproduced numerically ^32,34^. Furthermore, FEM approaches offer valuable insights into the elastic-plastic stress response of the film/substrate. Lichinchi et al., ^33^ used a combination of nano-indentation and FE-based analysis to understand the nano-mechanical behavior. However, during the operation cycle, the cracks may develop either at the SiCN-Si interface or within the SiCN thin film ^35^. These cracks typically initiate at the microscale level, highlighting the importance of comprehending the fracture behavior of the coating-substrate system at the micron length scale. Nevertheless, there is a lack of literature regarding understanding delamination in SiCN-Si coating systems. To this end, the main objective of this paper is to experimentally investigate the influence of applied load on different mechanical properties of CVD deposited SiCN thin film. Subsequently, we aim to validate a finite element (FE) model by comparing the simulated load-indentation response against the experimentally obtained results for various applied loads. The validated FE model will then be employed to simulate the fracture behavior of the CVD deposited SiCN thin film, providing valuable insights into its structural integrity and performance.

The rest of the paper is organized as follows: Section 2 presents the experimental setup overview and sample characterization. The FE-model and assumptions are explained in Section 3. Section 4 presents the results pertaining to experimental as well as FE simulation of nano-indentation, and fracture of the SiCN-Si system. The last section is dedicated to the future outlook and concluding remarks.

## Experiments and materials

### Sample preparation

**Fig. 1:**
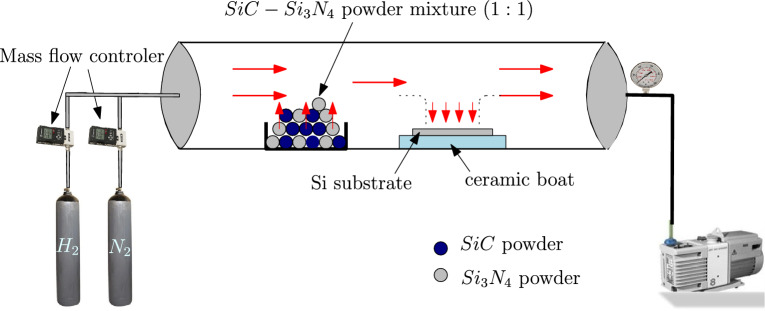
Schematic representation of experimental setup, atmospheric pressure chemical vapor deposition (APCVD).

In this study, a thin film of SiCN was deposited using a thermal CVD over a p-type c-Si (100) substrate at a constant temperature while maintaining constant flow rates of $\hbox {N}_{2}$ and $\hbox {H}_{2}$. The RCA cleaning protocol (Radio Corporation of America) was first used to clean the samples. Silicon carbide (SiC) and silicon nitride ($\hbox {Si}_{3}\hbox {N}_{4}$), with particle sizes of $\approx$ 100nm and $\approx$ 50nm, respectively and a mixture of $\hbox {N}_{2}$ and $\hbox {H}_{2}$ gas as precursor gases to produce the thin film on the substrate. The powder was stored in the ceramic boat and placed behind the substrate after being combined in a 1:1 ratio. The schematic internal arrangement of the CVD chamber is shown in Fig. 1. In order to exhaust the residual gases, the CVD chamber was pumped to subatmospheric pressure. The chamber’s base and process pressures were maintained at $3 \times 10^{-6}$ Torr and 300 mTorr, respectively. The carrier gas $\hbox {N}_{2}$ (99.99% pure) and precursor gas $\hbox {H}_{2}$ (99.99% pure) flow rates were both kept at 12 sccm, while the deposition temperature was maintained at 850$^{\circ }\,\hbox {C}$. For every sample, the heating and cooling rates were kept at $3^{\circ }\,\hbox {C/min}$ and ${5}^{\circ }\,\hbox {C/min}$, respectively.

### Sample characterization

At room temperature, the nano-indentation technique (TI 950, Hysitron Inc., USA with in-situ SPM (Scanning Probe Microscopy) imaging facility and standard Berkovich indenter tip) was used to analyze the mechanical characteristics of the SiCN thin film. The loading/unloading rate was 0.8 mN/s, aiming to maintain a quasi-static loading regime and minimize the influence of strain rate or thermal drift ^36^, and the maximum applied load ranged from 1 to 4 mN. It was ensured that the maximum penetration depth during indentation did not exceed 10% of the film thickness, which is in accordance with the Oliver & Pharr method. It was ensured that the maximum penetration depth during indentation did not exceed 10% of the film thickness, which is in accordance with the Oliver & Pharr method^36^. The tip area function was calibrated using a standard quartz sample, which corrects for tip rounding and ensures accurate contact area estimation^37,38^. Thermal drift was monitored using a standard post-unloading drift correction procedure. After each indentation cycle, the indenter was held in contact with the sample at a minimal load ($\sim$ 10% of maximum) for 60 seconds. The rate of depth change during this period was recorded and used to correct the displacement data across the full load-displacement curve. All tests were conducted after allowing the system to thermally stabilize for at least 45 minutes, and the recorded drift rates were below 0.05 nm/sec, which is within the acceptable range reported in the literature. This correction ensures the accuracy of mechanical property measurements, particularly at shallow indentation depth. At least 10 indentations were performed at each load level on different locations of the sample surface. The spacing between two consecutive indentations was maintained at at least 10 times the maximum indentation depth following the widely accepted guideline to minimize stress field overlap and substrate influence. This ensured that each indentation was independent and did not affect neighboring measurements, thereby preserving the accuracy and reliability of the mechanical property data ^39^. In order to analyze other mechanical characteristics of the SiCN thin film, the mean values of Hardness (H) and Young’s modulus (E) were taken into account. The coating thickness of SiCN thin films was measured using a Dektak profilometer (model no. Dektak V300) as $5.1\,\upmu \,\hbox {m}$.

**Fig. 2:**
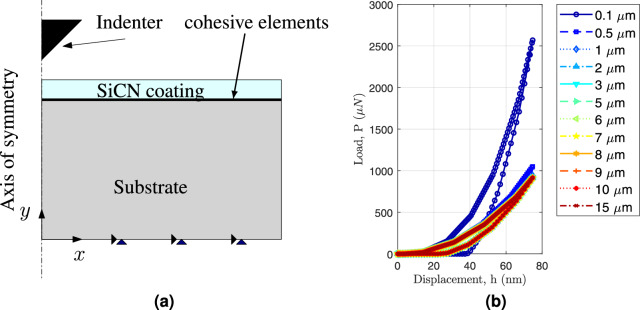
**a** Schematic representation of the domain and the boundary conditions of the nanoindentation process, **b** load-displacement response for varying thickness of the substrate.

## Finite element model

Figure 2a depicts the schematic representation of the finite element model employed for nanoindentation. The physical model is composed of SiCN coated (thickness $t_{\textrm{SiCN}}=5.1 \upmu \,\hbox {m}$) on Si substrate (thickness $t_{\textrm{Si}}=3\,\hbox {mm}$). To investigate the effect of substrate thickness on the load-displacement response, we performed finite element simulations by varying the coating thickness from $0.1\,\upmu \,\hbox {m}$ to 15$\upmu \,\hbox {m}$, while keeping all other material and loading parameters constant, as shown in Fig. 2(b). The results revealed that for coating thicknesses $\ge$ 2$\upmu \,\hbox {m}$, the load–displacement curves exhibited no significant variation, indicating a minimal influence of the substrate beyond this thickness threshold. Given that the coating thickness in our experiments was 5.1$\upmu \,\hbox {m}$, the simulation results confirm that the indentation response in our case is predominantly governed by the coating, with minimal or no contribution from the substrate.

The interface between the Si substrate and the SiCN coating is represented using cohesive elements with varying interfacial fracture resistance to mimic strong and soft SiCN-Si interfaces. A sharp Berkovich indenter composed of a diamond is employed to replicate the conditions of the nanoindentation experiment. The indenter features a semi-vertical angle of 70.3$^{\circ }$, which ensures a consistent relationship between the applied load and the depth of indentation while maintaining the same contact area^31^.

**Table 1:** Mechanical properties of the indenter, coating, and substrate materials^31^.

System & Material	Properties
Indenter: Diamond	(a) Young’s Modulus, E = 1141 GPa (b) Poisson’s ratio = 0.07
Coating: SiCN	(a) Young’s Modulus, E = 110 GPa (b) Poisson’s ratio = 0.2 (c) Hardness = 10 GPa
Substrate: Si	(a) Young’s Modulus, E = 187 GPa (b) Poisson’s ratio = 0.278 (c) Hardness = 25.5 GPa

The model is built upon the following assumptions:

- The indenter is considered perfectly rigid.
- The influence of friction between the SiCN film and the indenter is found to be negligible^31^. For the sake of simplicity, it is assumed that there is no friction between the indenter and the SiCN film’s top surface.
- A tie constraint is enforced between the bottom of the coating surface and the top of the cohesive zone surface. Another tie constraint is also established between the bottom of the cohesive zone surface and the top of the substrate.
- Surface-to-surface contact is provided between the bottom of the coating surface and the top of the substrate surface.

In this work, the commercial finite element package Abaqus^®^ ^40^ is utilized for the numerical simulation. The bottom edge is constrained in the $y-$direction. The film and substrate are both modeled using four-noded axisymmetric elements, CAX4. In the FE simulation, a highly refined mesh with an element size of 0.00003 mm was employed in the vicinity of the indenter to accurately capture the stress gradients. Whereas, a coarser mesh with an element size of 0.3 mm was utilized in the far-field regions to optimize the computational efficiency without compromising the accuracy of the localized deformation. The simulation is conducted in displacement control mode^41^. Therefore, for producing the load-displacement ($p-h$) curve, the vertical reaction is measured at the reference point on the indenter. Table 1 tabulates the material properties used for the indenter, substrate, and SiCN film. The SiCN coating is modeled as an elasto-plastic material, while the Si substrate is modeled with a linear elastic material. The cohesive zone model (CZM) parameters, such as cohesive strength and interfacial fracture energy, were not directly measured in this study. Instead, they were set using a trial-and-error approach, where the parameters were iteratively adjusted to achieve a reasonable agreement between the simulation results and qualitative experimental observations.

Cohesive elements are implemented using the COHAX4 cohesive material, which allows for the simulation of interface fracture or delamination through zero-thickness cohesive elements with an initial thickness of 0.00001 mm. These elements are inserted along the interface between the coating layer and the substrate. The COHAX4 elements incorporate interaction properties such as stiffness, damage initiation, and displacement-based damage evolution. The cohesive behavior is defined with stiffness values of 28 N/mm$^{3}$ in the normal direction and 14 N/mm$^{3}$ in each shear direction. Damage initiation follows the maximum nominal stress criterion (MAXS), with values of 0.075 N/mm$^{2}$ in the normal direction and 0.035 N/mm$^{2}$ in shear. Damage evolution is governed by a displacement value of 0.025mm. Surface energy for Mode I (0.0009375N/mm) and Mode II (0.0004375N/mm) is used. The crack path is predefined along this interface. A tie constraint is applied between the bottom surface of the coating layer and the top surface of the cohesive layer, as well as between the top surface of the substrate and the bottom surface of the cohesive layer. The cohesive surfaces act as the slave surfaces, while the top of the substrate and the bottom of the coating layer serve as the master surfaces surrounding the cohesive zone. Additionally, a surface-to-surface contact with surface behavior defined as pressure-overclosure = HARD is provided between the substrate and the coating layer, where the substrate is assigned as the master surface and the coating as the slave surface.

## Results and discussion

In this section, we begin by discussing the experimental findings regarding the influence of the maximum applied load on the mechanical properties of the SiCN thin film ( Section 4.1). Subsequently, we proceed to validate the Finite Element (FE) model for the nanoindentation process by comparing it to the experimental results. Finally, we present the results and analysis related to the delamination of the SiCN film in Section 4.2 using the validated FE model.

### Experimental results and FE-simulation

**Fig. 3:**
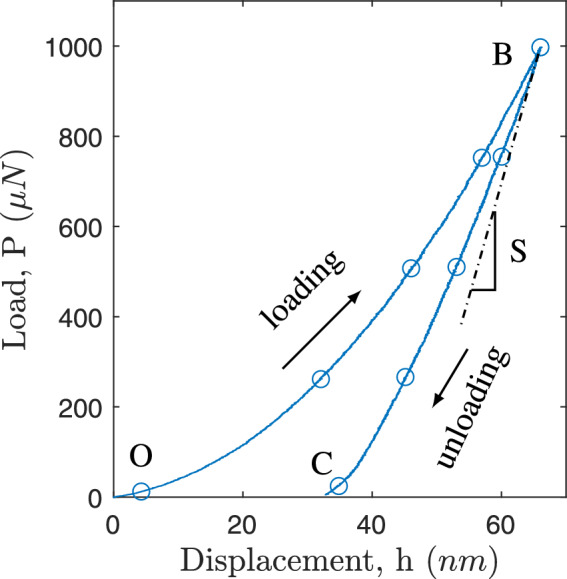
Schematic representation of a typical load-displacement response. Here, *h* is the indenter displacement, P is the reaction force, and S is the contact stiffness.

During the nano-indentation test, the Berkovich indenter (a diamond indenter with a three-sided pyramidal shape and high spatial resolutions) is continuously pushed into the specimen’s surface using mechanical force. The $P-h$ curve is produced by constantly recording the indentation depth (displacement) and applied force (load) of the indenter into the specimen surface, as illustrated in Fig. 3.

The gradual loading over the coating is represented by the section of the $P-h$ curve that runs from O to B, whereas the elastic unloading is represented by the piece of the $P-h$ curve that runs from B to C. The amount of energy lost overall while the coating was deformed is shown by the area under the $P-h$ curve for each indentation run. The $P-h$ curve follows the power-law expression given by the Oliver-Pharr approach:

1 $$\begin{aligned} P = \alpha (h_{max}-h_f)^{m} \end{aligned}$$

where *P* is the applied load, $h_f$ is the final displacement after complete unloading, $\alpha$ and *m* are material constants in $P-h$ relation to loading and unloading. In a thorough investigation of numerous materials, Oliver and Pharr established that the variation of the power law exponent is within the range of 1.2 $\le$ *m* $\le$ 1.6, which rejects the flat punch approximation ($m = 1$) and instead approaches to a paraboloid of rotation ($m = 1.5$). Oliver and Pharr were surprised by this outcome because the axisymmetric analogue of the Berkovich indenter is a cone ($m = 2$)^36^. The idea of “effective indenter shape”, described in full by Pharr and Bolshakov^42^, explained the inconsistency.

**Fig. 4:**
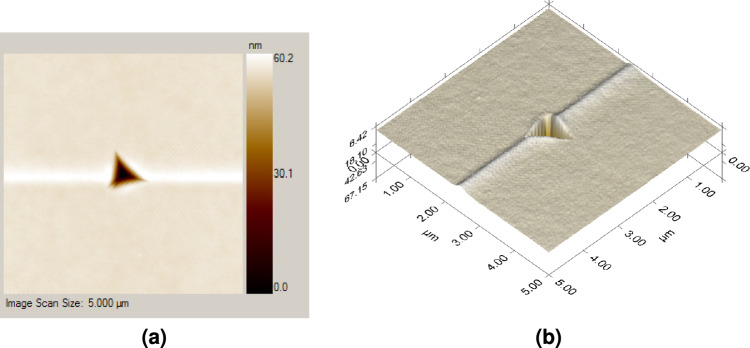
2D and 3D SPM micrograph of an indentation pattern at 4 mN peak load.

Ten different indenter penetration points were used to test each sample. An extra effort was taken when performing the nanoindentation test on each sample to limit the maximum penetration depth of the indenter to less than 10% of the total thickness of the coating to exclude the influence of substrate hardness completely (Fig. 4 shows a typical indent impression in 2D and 3D).

For analyzing nanoindentation load-displacement data, the Oliver and Pharr approach is used^43^. This method calculates hardness (H) and Young’s modulus (E) using the data from a single complete loading and unloading cycle. Hardness *H* is determined as

2 $$\begin{aligned} H = \frac{P_{\textrm{max}}}{A_{\textrm{max}}} \end{aligned}$$

where $P_{\textrm{max}}$ is the maximum indentation load over the coating, $A_{\textrm{max}}$ is the contact area of the indenter with the sample surface. For Berkovich tip, $A_{\textrm{max}}$ can be determined by assessing an experimentally calculated indenter shape function at the contact depth, $h_c$, which is given as

3 $$\begin{aligned} A_{\textrm{max}} = 24.56 h_c^2. \end{aligned}$$

The contact depth $h_c$ is determined from the $P-h$ curve as

4 $$\begin{aligned} h_c = h_{\textrm{max}} - h_s = h_{\textrm{max}} - \varepsilon \frac{P_{\textrm{max}}}{S} \end{aligned}$$

where $h_{\textrm{max}}$ is the maximum indentation depth, $\varepsilon$ is the geometric constant which is 0.76 for Berkovich indenter, and *S* is the unloading stiffness. The unloading stiffness *S* is defined as the slope of the unloading curve at the maximum penetration depth during the initial unloading stages as

5 $$\begin{aligned} S = |\frac{dP}{dh}|_{P = P_{\textrm{max}}} = 2 \beta \sqrt{\frac{A_{\textrm{max}}}{\pi }}E_r \end{aligned}$$

where $\beta$ is the indenter constant and is equal to 1.034 for Berkovich indenter and $E_r$ are the reduced modulus. The reduced modulus $E_r$ is given as

6 $$\begin{aligned} \frac{1}{E_r} = \frac{1-\nu ^2}{E} + \frac{1-\nu ^2_{id}}{E_{id}} \end{aligned}$$

where suffix *id* represents the indenter. The elastic modulus of the specimen *E* is given as

7 $$\begin{aligned} E = \frac{S \sqrt{\pi } E_{id} (1-\nu ^2) }{2 \sqrt{A_{\textrm{max}}} E_{id} -S \sqrt{\pi } (1-\nu _{id}^2)} \end{aligned}$$

**Fig. 5:**
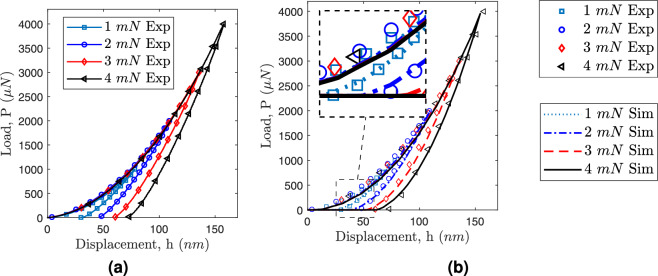
Load-indentation depth for the different maximum loads applied **a** experimental results, and **b** FE-simulation results compared against the experimental results.

Figure 5a shows the experimental $P-h$ curve for different applied loads. The loading curve follows the same path and returns back during the unloading process. The observed recovery displacement at the end of the unloading process suggests that the SiCN film exhibits greater resistance to recover from significant plastic deformation induced by higher maximum loads. The FE-simulation results show excellent agreement with the experimental results for both the loading and unloading process (see Fig. 5(b))

**Fig. 6:**
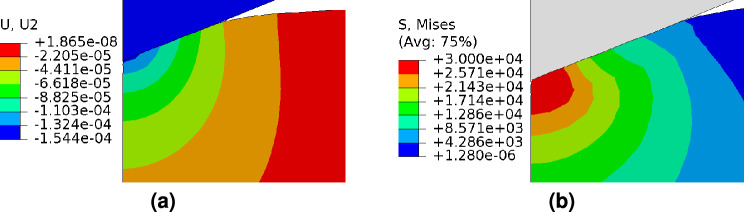
(a) Displacement profile $u_y$ at the end of loading step, and (b) Von-misses stress distribution around the indenter tip for different applied load 4 *mN*.

The SiCN is deformed during the nanoindentation process as the indenter tip descends. Moreover, as seen in Fig. 6(a), the deformation area is constrained close to the indenter tip. The maximum stress is also observed at the indenter tip (see Von-Misses stress distribution in Fig. 6(b)). At the indenter tip, the maximum stress reaches 30 GPa, exceeding the Yield stress $\sigma _y=$ 16.4 GPa to 18.2 GPa. Hence, the plastic deformation occurs around the indenter tip during the loading step. During the unloading step, the stress level will continue to go down, but this reduction will be constrained by the elastic recovery. The elastic recovery reduces with increasing maximum applied load, indicating that SiCN coating’s severe plastic deformation prevents elastic recovery.

**Fig. 7:**
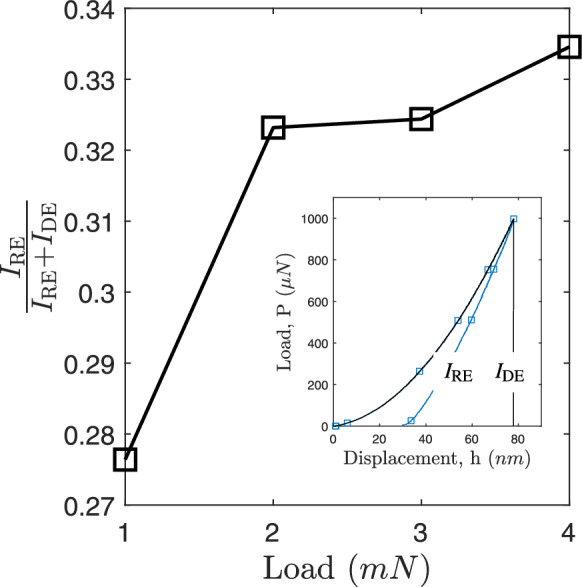
Recovery energy, $I_{Re}$ and total energy ($I_{Re}$ + $I_{De}$) ratio at different loads.

The values of the ratios between total energy $(I_{Re} / I_{De})$ and elastic recovery energy $I_{Re}$ for various applied loads are shown in Fig. 7. The region under the loading and unloading curve of nanoindentation can be used to compute $I_{De}$, which stands for the dissipated energy. The elastic behavior of thin films is shown by the $I_{Re}/(I_{Re} + I_{De})$ parameter. A decrease in the SiCN thin film’s plasticity is shown by an increase in the $I_{Re}/(I_{Re} + I_{De})$ parameter. As the applied load increased, the $I_{Re}/(I_{Re} + I_{De})$ value rose from 0.277 to 0.335, indicating an increase in the plasticity of the SiCN thin film.

### Fracture in SiCN-Si coated system

*In addition to finding hardness and elastic modulus, the nanoindentation technique can also be used to find the adhesion energy by creating well-defined areas of delamination. The nanoindentation-induced delamination enables the characterization of interfacial fracture by combining mechanics-based FE models *^44^. The experimentally validated FE model is now being utilized to acquire valuable insights into the fracture behavior of the SiCN film on a Si substrate. This understanding is derived through two specific tests: (a) the delamination and cracking of the SiCN layer during the nanoindentation process, and (b) the peel test. To achieve delamination of the SiCN coating, a strong or weak cohesive interface between the SiCN and Si substrate is employed, as explained in Section 3. The thickness of the SiCN coating is set as 5.1$\upmu \,\hbox {m}$. This specific thickness is primarily chosen to observe the delamination process.

**Fig. 8:**
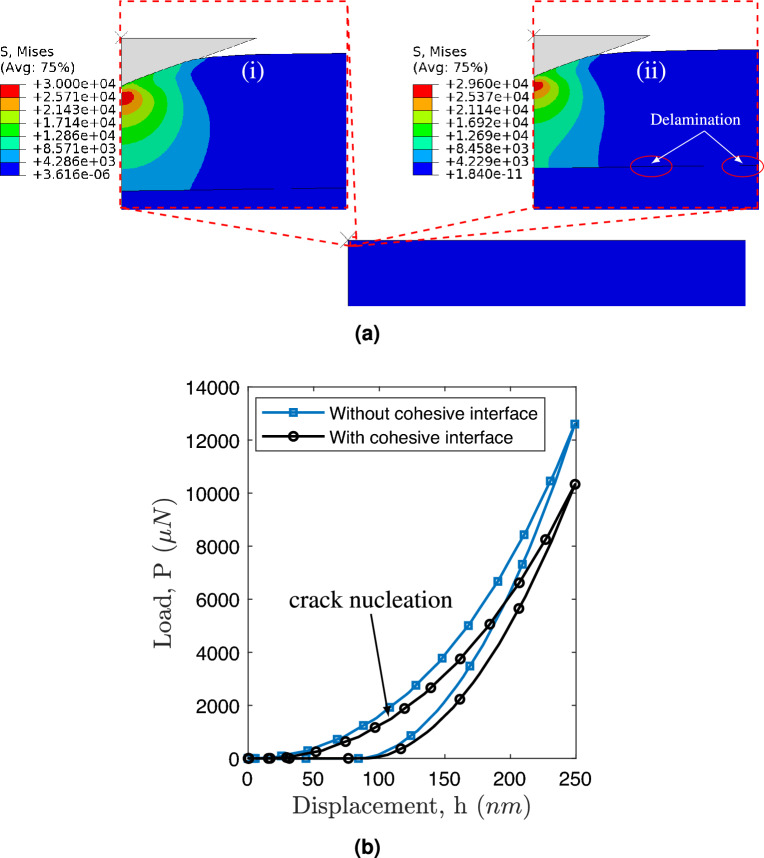
Cohesive delamination of SiCN thin film during nanoindentation test **a** (i) no delamination, and (ii) with delamination, and **b** Load-displacement curve with and without a cohesive interface.

Figure 8a depicts the delamination behavior during the nanoindentation test. It is observed that delamination is effectively prevented when a strong cohesive interface is present, as shown in Fig. 8a(i). However, in the case of a weak interface, the crack nucleates along the interface at an indent displacement $h=$ 110nm (see Fig. 8a(ii)). Furthermore, the crack widens during the unloading process. These findings align with the experimental results for Si$_3$N$_4$ film^44^. * Kleinbichler et. al,* ^44^ *also observed multiple interfacial cracks between *Si$_3$N$_4$ *and borophosphosilicate glass (BPSG) along with crack kinking. However, our numerical simulation did not show such phenomena, as propagation is restricted to cohesive elements in the cohesive zone model. This limitation can be addressed using Fracture models such as the continuum damage model* ^45^, * the phase-field model* ^46^, * which is scope for future study.* The simulation results indicate that sufficient interfacial strength is required to prevent the delamination of the SiCN coating during the nanoindentation process. The overall fracture toughness also decreases due to the delamination of the interface, as shown in Fig. 8b.

**Fig. 9:**
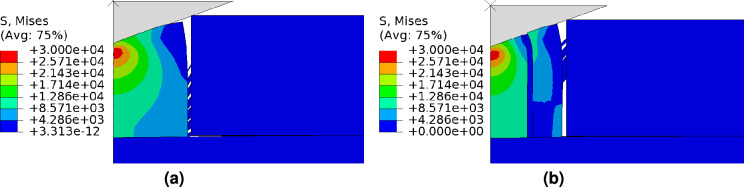
Cohesive delamination and cracking of SiCN coating: **a** one vertical crack with the cohesive interface, and **b** two vertical cracks with the cohesive interface.

**Fig. 10:**
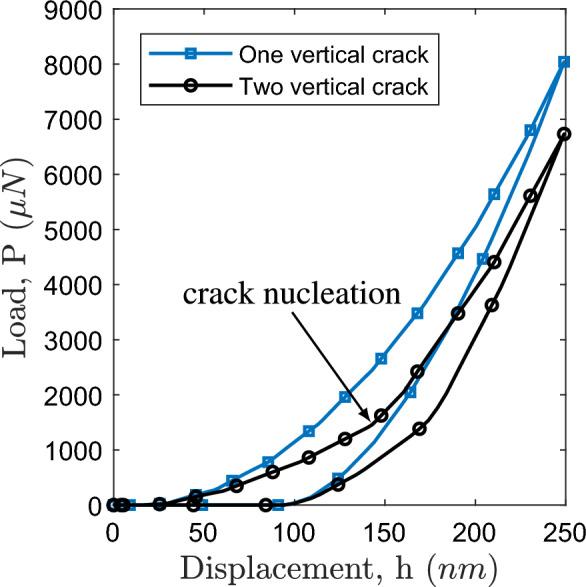
Load-displacement curve for one vertical crack, and two vertical cracks.

*In order to understand the behavior of crack kinking after the crack nucleation along the interface,* we introduced cohesive elements within the SiCN film. These cracks were strategically placed to simulate potential failure sites. Initially, a single crack was inserted into the SiCN films, followed by the application of a loading force. The crack initiation primarily occurred along the interface under the applied load and subsequently propagated within the film itself (refer to Fig. 9(a)). Then we place two cracks in the SiCN film. In this case, the interface between SiCN and Si is also delaminated and on further application of load, the second crack nucleated, as shown in Fig. 9(b). The overall fracture toughness also decreases in the presence of cracks as depicted using the load-displacement response (see Fig. 10).

**Fig. 11:**
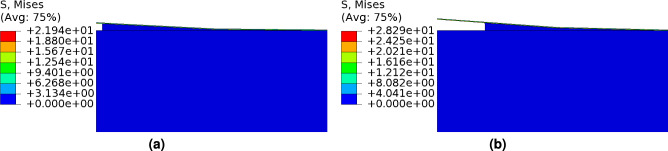
Peel test: **a** crack initiation along the interface, and **b** subsequent crack propagation.

**Fig. 12:**
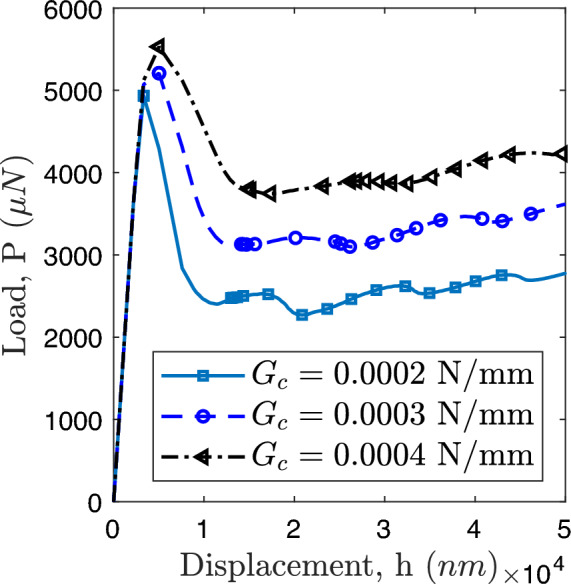
Peel test: load-displacement response for different critical fracture roughness $G_c$ of interface.

In order to conduct a peel test, a vertical upward displacement is now applied to the left edge of the SiCN coating. At the critical stress, crack nucleation occurs along the interface, as depicted in Fig. 11a. As the applied load continues to increase, the nucleated crack propagates, as demonstrated in Fig. 11b. Notably, the load required to propagate the crack decreases following crack nucleation as evidenced by the findings in Fig. 12. Moreover, the critical fracture energy of the interface plays a significant role in the interface debonding. The increase in this parameter correlates with an increased load required for crack nucleation.

## Conclusions

This work focuses on investigating the effect of the maximum load on the mechanical properties of the SiCN thin film using nano-indentation experiments. Additionally, the experimental results validate the FE model implemented in the commercial finite element package Abaqus. The experimentally validated FE model is then used to understand the delamination of the SiCN-coated specimen during nano-indentation. The analysis of delamination through both nanoindentation and peel testing allowed for a comprehensive assessment of the fracture behavior. Different fracture modes were observed in each case, highlighting the complexity and diversity of the delamination process. This understanding could facilitate the design and optimization of SiCN-coated systems, enabling enhanced mechanical performance and reliability. By expanding the knowledge of fracture mechanisms and their dependence on applied loads, it can be used to develop robust and durable coating systems for various industrial applications.

## Data Availability

The datasets generated during and/or analysed during the current study are available from the corresponding author on reasonable request.
